# Integrative Molecular and Immune Profiling in Advanced Unresectable Melanoma: Tumor Microenvironment and Peripheral PD-1+ CD4+ Effector Memory T-Cells as Potential Markers of Response to Immune Checkpoint Inhibitor Therapy

**DOI:** 10.3390/cancers17122022

**Published:** 2025-06-17

**Authors:** Manuel Molina-García, María Jesús Rojas-Lechuga, Teresa Torres Moral, Francesca Crespí-Payeras, Jaume Bagué, Judit Mateu, Nikolaos Paschalidis, Vinícius Gonçalves de Souza, Sebastian Podlipnik, Cristina Carrera, Josep Malvehy, Rui Milton Patricio da Silva-Júnior, Susana Puig

**Affiliations:** 1 Institut d’Investigacions Biomèdiques August Pi I Sunyer (IDIBAPS), 08036 Barcelona, Spain; manmolina@recerca.clinic.cat (M.M.-G.); tetorres@recerca.clinic.cat (T.T.M.); fjcrespi@recerca.clinic.cat (F.C.-P.); bague@recerca.clinic.cat (J.B.); mateu@recerca.clinic.cat (J.M.); podlipnik@clinic.cat (S.P.); ccarrera@clinic.cat (C.C.); jmalvehy@clinic.cat (J.M.); patricioda@recerca.clinic.cat (R.M.P.d.S.-J.); 2 Facultat de Medicina i Ciències de la Salut, Universitat de Barcelona (UB), c. Casanova 143, 08036 Barcelona, Spain; 3 Melanoma Unit, Dermatology Department, Hospital Clinic of Barcelona, 170 Villarroel, 08036 Barcelona, Spain; 4 Otorhinolaryngology Department, Hospital Clínic de Barcelona, CIBERES, IDIBAPS, Universitat de Barcelona, 08036 Barcelona, Spain; mrojas@clinic.cat; 5 Centre of Biomedical Research on Rare Diseases (CIBERER), Instituto de Salud Carlos III, 08036 Barcelona, Spain; 6 Biomedical Research Foundation, Academy of Athens (BRFAA), 4 Soranou Efesiou St., 11527 Athens, Greece; npaschal@bioacademy.gr; 7 Molecular Oncology Research Center, Barretos Cancer Hospital, Barretos 14784-400, SP, Brazil; vinicius.gs2110@edu.hospitaldeamor.com.br; 8 Department of Cell and Molecular Biology, Faculty of Medicine of Ribeirão Preto, University of São Paulo (FMRP-USP), Ribeirão Preto 14049-900, SP, Brazil

**Keywords:** melanoma, tumor microenvironment, immunophenotyping, immune checkpoint inhibitors, gene expression profiling, biomarkers, interferon-gamma, programmed cell death 1 receptor, CTLA-4, progression-free survival

## Abstract

**Background/Objectives:** Immune checkpoint inhibitors (ICIs) have revolutionized advanced melanoma treatment, yet many patients fail to achieve sustained clinical benefit. Several biomarkers, including tumor microenvironment (TME) signature, PD-1/PD-L1 expression, and IFN-γ signaling, have been proposed. However, robust predictive markers remain elusive. This study aimed to identify molecular markers of response by analyzing tumor and peripheral immune signatures. **Methods:** This study analyzed 21 advanced melanoma patients treated with ICIs. Formalin-fixed, paraffin-embedded tumors underwent RNA-sequencing targeting 1392 immuno-oncology probes. Genes significantly associated with progression-free survival (PFS) by log-rank test underwent hierarchical clustering analysis (HCA). Differential expression and xCell analyses were then performed on the resulting clusters. Cox multivariate analysis was applied to identify independent PFS predictors. Pre-treatment peripheral blood mononuclear cells were analyzed by mass cytometry, followed by FlowSOM and UMAP clustering. **Results:** Fifty-five genes significantly associated with PFS identified two molecular clusters via HCA. Cluster A demonstrated prolonged PFS (59.4 vs. 2.4 months, *p* = 0.0004), while Cluster B was characterized by downregulated IFN-γ signaling, antigen presentation pathways, and reduced immune score. Multivariate Cox analysis confirmed molecular cluster as an independent PFS predictor (*p* < 0.001). Mass cytometry revealed higher frequencies of circulating PD-1+ CD4+ effector memory (EM) T subpopulations among responders. **Conclusions:** This study highlights the potential role of molecular and immune profiling in predicting ICI response in advanced melanoma. The identification of distinct molecular clusters underscores significant TME heterogeneity, with immune-cold tumor clusters associated with poorer outcomes. Furthermore, circulating PD-1+ T subpopulations emerged as potential markers of ICI response, suggesting their value in improving patient stratification.

## Simple Summary

Advanced melanoma is an aggressive skin cancer that has spread and cannot be surgically removed. Immune checkpoint inhibitors (ICIs) have significantly improved survival for some patients by helping the immune system recognize and attack cancer cells. However, reliable methods to predict which patients will benefit are still lacking. This study investigated whether analyzing both tumor and blood samples before treatment could help identify patients most likely to benefit from ICIs. By studying gene activity in tumors and immune cell types in the blood, we found that patients with low immune activity in their tumors had poorer outcomes. Conversely, those with higher levels of a specific immune cell type in their blood—PD-1+ effector memory CD4+ T-cell—tended to respond better to treatment. These findings suggest that combining both tumor and blood analysis could support more personalized and effective treatment decisions for patients with advanced melanoma.

## 1. Introduction

Immune checkpoint inhibitors (ICIs) have revolutionized the treatment landscape for advanced melanoma, offering durable responses and significantly improved overall survival in a subset of patients. However, clinical outcomes remain heterogeneous, with 40–60% of patients failing to achieve sustained clinical benefit from ICIs [1,2,3,4,5,6]. Among non-responders, progression-free survival (PFS) is typically limited to 2–6 months, highlighting the need for robust predictive biomarkers and personalized treatment strategies [2,6,7].

Advanced melanoma exhibits substantial tumor heterogeneity, with tumor-intrinsic and microenvironmental factors shaping ICI response. Notably, high tumor mutational burden (TMB) [8], increased tumor-infiltrating lymphocytes (TIL) [9,10,11], elevated PD-1/PD-L1 expression [2,12,13,14], and IFN-γ signature [15,16] have been generally correlated with improved immunotherapy responses [11,17]. TILs have been widely associated with improved responses to ICIs due to their ability to recognize and attack tumor cells [9]. Their presence, particularly of CD8+ T-cells, reflects an active immune response, yet tumors often evade immune attack through PD-1/PD-L1-mediated adaptive resistance [9]. High tumoral PD-L1 expression has been proposed as a predictive biomarker for response to anti-PD-1 therapy, with patients exhibiting PD-L1-positive tumors showing superior treatment outcomes compared to those with PD-L1-negative tumors [18,19]. Furthermore, TILs are often accompanied by an IFN-γ signature, a key immunoregulatory pathway that enhances antigen presentation, promotes T-cell recruitment, and boosts cytotoxic activity. However, sustained IFN-γ signaling can lead to adaptive immune resistance by inducing the expression of immune checkpoint molecules, ultimately dampening the antitumor response [15,20,21]. Moreover, inadequate antigen presentation within the tumor can drive T-cell anergy, a state characterized by defective T-cell receptor signaling, diminished proliferative capacity, and reduced production of effector cytokines. These anergic T-cells fail to exert cytotoxic functions and are refractory to further stimulation, thereby compromising the efficacy of ICIs. However, defining these cells is challenging due to the absence of unique surface markers [22,23,24,25,26]. This complex regulatory landscape underscores the intricacy of tumor-immune crosstalk and highlights the need for comprehensive, biomarker-driven strategies to maximize the efficacy of ICIs in advanced melanoma. 

While tumor tissue analyses provide key insights into ICI response, they may not fully capture the dynamic and evolving nature of antitumor immune responses. In this context, peripheral blood profiling has emerged as a practical approach. Responders to ICI therapy exhibit higher-circulating PD-1+ CD8+ T-cells before treatment, as well as higher percentages of circulating CD8+ effector memory T-cells before treatment [27]. Additionally, the reinvigoration of Ki-67+ circulating exhausted-phenotype CD8+ T-cells in relation to pretreatment burden correlates with response [28], whereas treatment failure links to an imbalance between T-cell reinvigoration and tumor burden [28]. Beyond T-cells, other immune cells populations can also influence outcomes [29].

Despite these advances, many questions remain regarding the interplay between tumor and immune factors in driving ICI response. Further studies integrating molecular and immunological data are critical to refine predictive models.

This study aimed to deepen the understanding of RNA-based tumor markers and cytometric profiles of peripheral blood mononuclear cells (PBMCs) associated with ICI response by examining molecular and immune signatures in patients with advanced, unresectable melanoma. Using both high-throughput RNA expression profiling and mass cytometry, we sought to characterize the tumor and immune landscapes related with immunotherapy response and identify clinically relevant markers to enhance patient stratification and therapeutic decision-making. This study identified distinct molecular clusters associated with immunotherapy response, where immune suppression and cell cycle dysregulation defined poor responders. Moreover, specific peripheral T-cell subsets, including PD-1+ effector memory (EM) CD4+ T-cells, emerged as potential markers of immunotherapy outcomes. 

## 2. Materials and Methods

### 2.1. Study Design, Patients, and Inclusion Criteria

This prospective study, performed at the Hospital Clinic of Barcelona (HCB) and approved by the HCB Ethics Committee (approval number HCB/2018/1074), enrolled advanced melanoma patients eligible for ICI therapy, including anti-PD-1 or a combination of anti-PD-1 and anti-CTLA-4. Inclusion criteria included signed informed consent and ICI eligibility according to clinical guidelines. Patients with prior ICI therapy, autoimmune disorders requiring immunosuppression, active infections (e.g., HIV, hepatitis B/C), or lacking both pre-treatment formalin-fixed paraffin-embedded (FFPE) biopsies and pre-therapy peripheral blood samples were excluded. FFPE primary tumors or, when unavailable, first metastatic sample obtained prior to ICI were analyzed. For the same patient cohort, PBMCs were prospectively collected before immunotherapy initiation. The timepoint of biopsies is shown in Supplementary Figure S1. PFS was measured from the start of immunotherapy until the last follow-up (1 December 2024) or until progression defined by the Response Evaluation Criteria in Solid Tumors (RECIST) [30]. Responders were defined as those achieving Complete Response (CR) or Partial Response (PR), while non-responders were classified as those with stable disease or progressive disease (PD). 

Demographic, clinical, and histopathological data—including ulceration, mitotic index, and BRAF mutations—were obtained from medical records (Supplementary Table S1). Melanoma staging followed the American Joint Committee on Cancer (AJCC) 8th edition guidelines [31]. 

An extended version of the methodology, including subsequent sections, is available in the Supplementary Information.

### 2.2. Tumor Sample Preparation, Library Construction, Sequencing, and Data Processing

Tumor samples were collected during surgery and then underwent an exhaustive histopathological and immunohistochemical analysis. Hematoxylin and eosin (H&E) staining was utilized to accurately identify tumor regions by an experienced pathologist. FFPE tissue blocks were cut into 5 µm sections, with microdissected tumor areas ranging from 12 to 30 mm^2^. HTG EdgeSeq technology was employed on the samples using the Precision Immuno-Oncology Panel (1392 probes, HTG, Tucson, AZ, USA) for quantitative mRNA expression profiling. All steps—including sample processing, library preparation, and sequencing—were performed according to the manufacturer’s protocol. Sequencing was conducted on an Illumina NextSeq 550 platform (Illumina, San Diego, CA, USA). 

### 2.3. Survival Analysis

Survival analyses were conducted using log-rank tests to assess the association between gene expression levels and PFS. Patients were divided into high and low expression groups based on the median counts per million (CPM) values. Kaplan–Meier curves compared the PFS between groups, with disease progression as the event of interest and PFS measured in months. 

Cox regression analyses examined associations between clinical and molecular factors and PFS. Initially, a univariate analysis assessed each variable independently, followed by a multivariate model adjusting for confounders, to identify independent immunotherapy response markers. Forest plots illustrated hazard ratios (HRs) and 95% confidence intervals (CIs). All analyses were performed in RStudio (v.2023.06.0+421), considering significance at *p* < 0.05. 

### 2.4. Hierarchical Clustering Analysis (HCA)

Gene expression raw counts (RCs) from genes significantly associated with PFS were analyzed. First, a DESeqDataSet (dds) (DESeq2 v.1.38.3) object was created, and CPM values, log_2_CPM transformation, and Z-scores were calculated for sample normalization. An HCA of the samples was conducted using Pearson correlation with average linkage. The ComplexHeatmap package (v.2.15.4) was used for heatmap visualization, integrating clinical annotations. A principal component analysis (PCA) on rlog-transformed data visualized sample separation. Finally, the cluster stability was assessed through pvclust (v.2.2-0) bootstrapping. 

### 2.5. Differential Expression Analysis (DEA)

A DEA was performed between clusters, using the group with better PFS as the reference. Differentially expressed genes (DEGs) were identified using DESeq2 (v.1.38.3), defining significance as adjusted *p*-value (*p*adj) < 0.05, calculated using Benjamini–Hochberg correction for multiple testing. Volcano plots highlighted genes with log_2_FoldChange ≥1 or ≤ −1 and *p*adj < 0.05 as upregulated or downregulated, respectively.

### 2.6. Gene Set Enrichment Analysis (GSEA)

GSEA was conducted to identify enriched pathways associated with differential expression between clusters. Probes lacking direct correspondences or linked to non-unique genes were reannotated or excluded (Supplementary Table S2). Using a curated panel of 1378 genes ranked by differential expression significance, enriched biological processes were assessed through the Gene Ontology (GO) Biological Process (BP) database, applying Benjamini–Hochberg correction (*p*adj ≤ 0.05). 

### 2.7. Immune, Stroma, and Tumor Microenvironment Signatures

The xCell algorithm [32] (v.1.1.0) characterized 19 immune and 4 stromal populations across clusters. Enrichment scores were calculated from normalized counts using xCell analysis with spillover correction. Comparisons between clusters were performed using *t*-tests (Student’s or Welch’s) for normally distributed data and the Mann–Whitney U test otherwise, with normality and variance homogeneity assessed by Shapiro–Wilk and Bartlett’s tests, respectively. 

### 2.8. Blood Sample Preparation, PBMCs Mass Cytometry Staining and Analysis

Blood samples were collected in acid–citrate–dextrose (ACD) collection tubes, and PBMCs were isolated using Ficoll gradient centrifugation and cryopreserved in liquid nitrogen. For analysis, PBMCs were thawed, washed, blocked, and stained with the Maxpar Direct Immune Profiling Assay (MDIPA, Standard BioTools, South San Francisco, CA, USA) plus 10 additional antibodies (Supplementary Table S3). The cells were then fixed, permeabilized, labeled with an iridium-based intercalator, and stored at −80 °C.

Before acquisition on the Helios mass cytometer (Standard BioTools, South San Francisco, CA, USA), the cells were thawed, washed, mixed with EQ Four Element Calibration Beads for normalization, and acquired using the MDIPA template. Data were normalized, exported as Flow Cytometry Standard (FCS) files, and processed in FlowJo^TM^ (v.10.8) using gaussian parameters [33]. Live singlet CD45+ cells were selected for further analysis (Supplementary Figure S2). An R-based pipeline integrated the Flow Self-Organizing Map algorithm (FlowSOM v.2.11.2), uniform manifold approximation and projection (UMAP uwot v.0.2.2), and the CATALYST package (v.1.26.1) for clustering [34]. Statistical significance was assessed using Wilcoxon tests with Benjamini–Hochberg correction (*p* ≤ 0.05). A receiver-operating characteristic (ROC) curve analysis was then performed in R using the pROC package (v.1.18.5), and the area under the curve (AUC) was calculated to quantify predictive performance for immunotherapy response. The optimal cutoff point for cell abundance was then identified with the Youden index (J = sensitivity + specificity − 1), selecting the threshold that maximizes the combined sensitivity and specificity for predicting immunotherapy response.

### 2.9. Statistical Analysis

Continuous variables were assessed for normality using the Shapiro–Wilk test. Normally distributed data were presented as means (standard deviations), while non-normally distributed data were expressed as medians (interquartile ranges, IQRs). Survival times were reported as medians (IQRs). Categorical variables were summarized as frequencies and percentages, then analyzed using a Chi-square (χ^2^) or Fisher’s exact test, as appropriate. All tests were two-tailed, with a 0.05 alpha level for statistical significance. Analyses were conducted using the R language (v4.2.3) in RStudio (v2023.06.0+421).

## 3. Results

### 3.1. Patient Demographics and Clinical Characteristics

A total of 21 non-surgical metastatic melanoma patients treated with ICI were involved in the study, including anti-PD-1 monotherapy or a combination of anti-PD-1 and anti-CTLA-4 therapy. However, two patients were excluded from the HTG-sequencing analysis and four patients from the mass cytometry analysis due to sample quality-control (QC) issues. 

The mean age at start of immunotherapy was 69.1 years (IQR: 15.2 years), with 38.1% (*n* = 8) of the cohort being female. At the start of immunotherapy, the distribution of staging was as follows: IIIB (*n* = 1; 4.8%), IIID (*n* = 2; 9.5%), IVM1a (*n* = 8; 38.1%), IVM1b (*n* = 5; 23.8%), IVM1c (*n* = 3; 14.3%), and IVM1d (*n* = 2; 9.5%). The median PFS was 28.7 months (IQR: 56.7) (Supplementary Tables S1 and S4). The estimated PFS rates at 1 year, 3 years, and 5 years were 52.4% (95% CI: 34.8–78.8), 42.9% (95% CI: 26.2–70.2), and 42.9% (95% CI: 26.2–70.2), respectively. 

### 3.2. Histopathological and Tumoral Molecular Findings

Of the 19 FFPE blocks analyzed, 15 (78.9%) were primary tumors and 4 (21.1%) cutaneous metastases. Primary tumors had a median Breslow thickness of 4 mm (IQR: 2.85), comprising five superficial spreading melanomas (SSM), five nodular melanomas (NM), one mucosal lentiginous melanoma (MLM), one acral lentiginous melanoma (ALM), and three acral nodular melanomas (ANM).

Histopathological analysis of all tumors showed a median mitotic rate of seven mitoses per mm^2^ (IQR: 6), with ulceration present in 73.7% (*n* = 14) of cases, and 42.1% (*n* = 8) being amelanotic. Cell morphology was predominantly epithelioid in 78.9% (*n* = 15), 10.5% (*n* = 2) were plasmacytoid, 5.3% (*n* = 1) microcytic/plasmacytoid, and 5.3% (*n* = 1) fusiform (Supplementary Table S1).

### 3.3. Log-Rank Analysis Identifies Candidate Genes Associated with PFS

A log-rank test applied to the high-throughput gene expression panel identified 55 candidate genes significantly associated with PFS (*p* < 0.05; Supplementary Table S5). Kaplan–Meier survival plots for the top 10 candidate genes with the lowest *p*-values are presented in Supplementary Figure S3. 

### 3.4. Hierarchical Clustering Analysis Identifies Molecular Subgroups Associated with PFS

**Figure 1:**
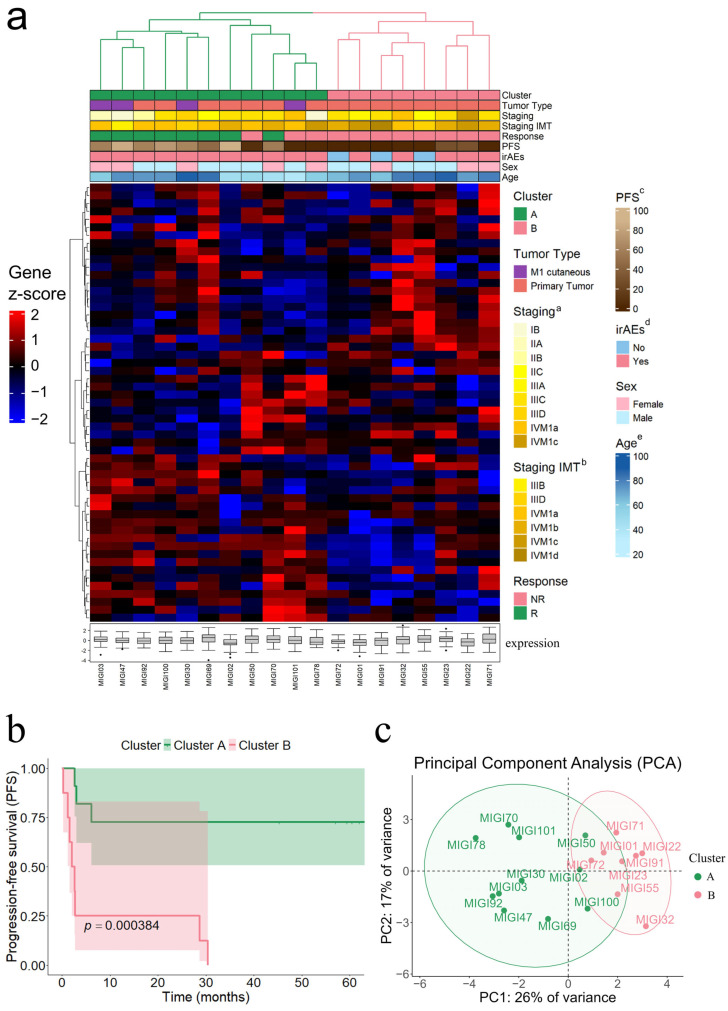
Hierarchical clustering analysis (HCA), Kaplan–Meier survival analysis, and principal component analysis. (**a**) Heatmap illustrating HCA based on the expression of 55 genes identified as significant in the log-rank test. Gene expression values were normalized as z-scores, represented on a blue-to-red scale for low to high expression. Boxplots beneath each sample indicate median expression, interquartile range, and outliers. Clustering was conducted using Pearson correlation and average linkage, with clinical annotations displayed above the heatmap. (**b**) Kaplan–Meier survival curves demonstrating progression-free survival differences between Clusters A and B identified through HCA. The *p*-value reflects the statistical significance of the survival differences between the two clusters. (**c**) Principal component analysis (PCA) of gene expression for the 55 significant genes, highlighting sample grouping into Clusters A and B. Shaded ellipses represent 95% confidence intervals for each cluster. ^a^: Staging: Staging at diagnosis; ^b^: Staging IMT: Staging at the start of immunotherapy; ^c^: PFS: progression-free survival; ^d^: irAEs: immune-related adverse events; ^e^: Age: age at the start of immunotherapy.

HCA based on 55 candidate genes from log-rank analysis revealed tumoral molecular heterogeneity, identifying two major clusters (Figure 1a).

Cluster A was enriched with patients exhibiting better PFS (median PFS: 59.4 months; IQR: 42.2), and Cluster B was characterized by significantly worse PFS (median PFS: 2.4 months; IQR: 7.7; *p* = 0.0004) (Figure 1b). Within each main cluster two subdivisions were identified, resulting in A1 and A2 within Cluster A, and B1 and B2 within Cluster B. Subcluster A1 demonstrated the most favorable PFS (median PFS: 61.2 months; IQR: 11.2), while B1 represented the group with the poorest PFS (median PFS: 1.6 months; IQR: 1.5; *p* = 0.0007) (Supplementary Figure S4). Subclusters A2 and B2 included fewer patients and exhibited more heterogeneous gene expression patterns.

Bootstrap analysis confirmed clustering stability and robustness with support values of 83 and 81 for the main branches of Clusters A and B, respectively (Supplementary Figure S5). PCA corroborate the clusters, with principal component 1 (PC1) and principal component 2 (PC2) explaining 26% and 17% of variance, respectively. The PCA plot demonstrated a distinct separation between Clusters A and B along PC1 (Figure 1c). 

### 3.5. Clinical Relevance of Molecular Signature Clusters in Response to Immunotherapy

Patients in Cluster B exhibited significantly shorter PFS compared to those in Cluster A (*p* = 0.0018), whereas Cluster A showed a significantly higher proportion of responders (*p* = 0.0034). However, no significant differences were found between clusters regarding immune-related adverse events (irAEs) (*p* = 0.0578), age at diagnosis (*p* = 0.0677), age at start of immunotherapy (*p* = 0.0716), tumor type (*p* = 0.1032), staging at diagnosis (*p* = 0.8978), staging at the start of immunotherapy (*p* = 0.3068), or sex (*p* = 1) (Table 1). 

**Figure 2:**
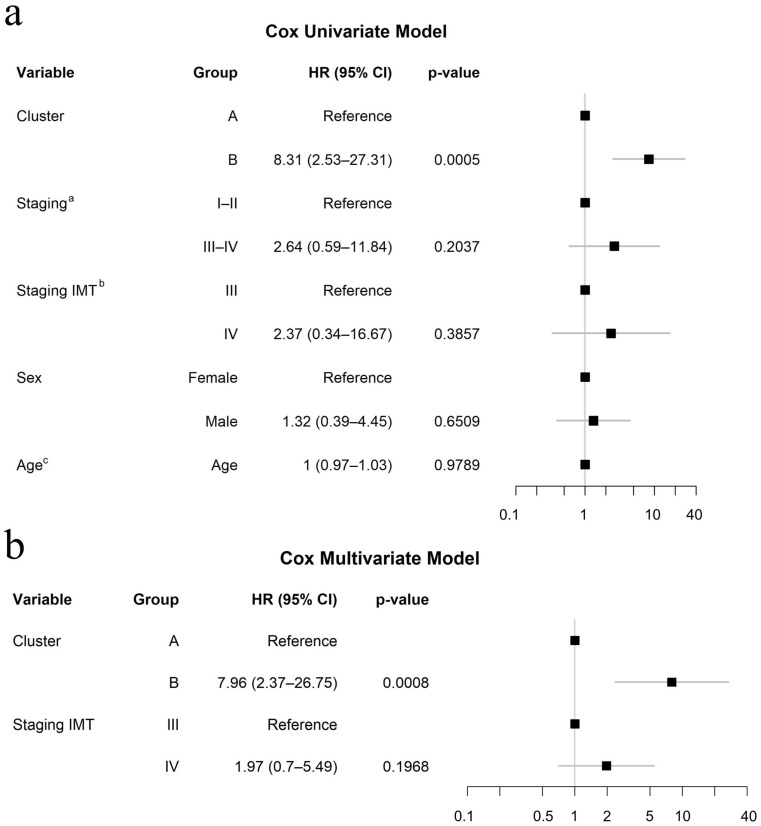
Univariate and multivariate Cox regression forest plots: (**a**) Univariate Cox regression analysis illustrating hazard ratios (HRs) with 95% confidence intervals for individual predictors of progression-free survival (PFS). Each HR represents the relative risk of progression associated with a given variable, with values greater than 1 indicating an increased risk and values less than 1 suggesting a potential protective effect. (**b**) Multivariate Cox regression model incorporating key predictors of PFS, adjusted for potential confounders. The included variables are Cluster and Staging IMT (staging at the start of immunotherapy). HRs and *p*-values were computed using robust standard error. The x-axis represents a logarithmic scale of HR. ^a^ Staging: Staging at diagnosis; ^b^ Staging IMT: Staging at the start of immunotherapy; ^c^ Age: age at the start of immunotherapy.

A univariate Cox proportional hazards model evaluated the impact of various factors on PFS, including cluster, staging at diagnosis, staging at the start of immunotherapy, sex, and age at the start of immunotherapy. Among these, only the variable cluster (*p* = 0.0005) was significantly associated with PFS outcomes (Figure 2a). In a subsequent multivariate model, only cluster remained an independent predictor of PFS. Specifically, Cluster B was strongly associated with shorter PFS (HR = 7.96, 95% CI: 2.37–26.75; *p* < 0.001) (Figure 2b).

### 3.6. Immune Suppression and Cell Cycle Pathway Alterations Have the Capacity to Define Molecular Differences Between Clusters

**Figure 3:**
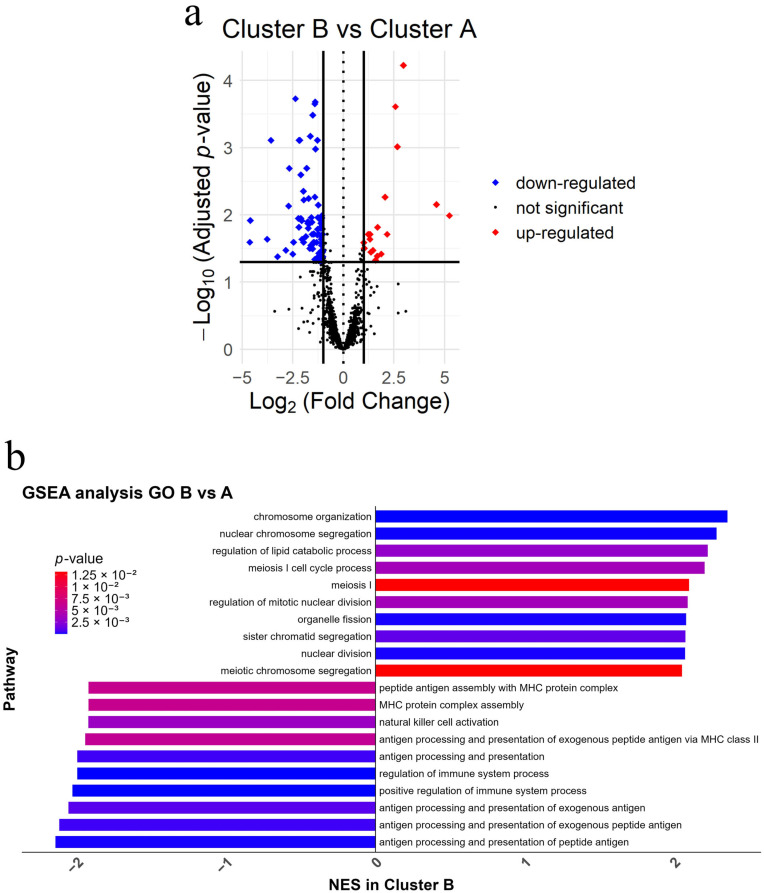
Differential gene expression, Venn diagrams, and gene set enrichment analysis (GSEA). (**a**) Volcano plots depicting differentially expressed genes (DEGs) between Cluster B and Cluster A. Significantly upregulated genes are highlighted in red, downregulated genes in blue, and non-significant genes in black. Significance was determined using *p*-adjusted < 0.05 and a fold-change threshold of ±1; (**b**) bar plot displaying the top 10 positively and negatively enriched pathways from GSEA using gene ontology (GO) terms in the B vs. A comparison. Bars represent normalized enrichment scores (NES), with pathway significance visualized using a color gradient for *p*-values.

To elucidate the molecular mechanisms underlying the differences between clusters, a DEA was performed. A total of 113 DEGs were identified between Clusters B and A, with 24 upregulated and 89 downregulated in Cluster B (*p*adj < 0.05) (Figure 3a and Supplementary Table S6). Given that the IFN-γ signature has been widely recognized as a predictor of response to ICI in melanoma [35], we further explored its involvement in our dataset. Filtering the genes from the HTG panel using the HALLMARK_INTERFERON_GAMMA_RESPONSE gene set (200 genes) from MSigDB (Molecular Signatures Database, Broad Institute), revealed 19/105 genes (18.1%) overlapped with the DEGs identified between Clusters B and A; 18/19 genes (94.7%) were downregulated, while *PTGS2* was upregulated in Cluster B. 

To corroborate these findings, an additional IFN-γ signature (10 genes) expanded with an immune-related gene signature (Preliminary expanded immune signature; PEI) previously associated with immunotherapy response in melanoma was examined. Specifically, the PEI signature (28 genes included) proposed by Ayers et al. (2017) [15] was analyzed, and all 28 genes were included in the HTG panel. A substantial overlap with DEGs was observed, with 15/28 genes (53.6%) downregulated in Cluster B, further supporting its association with poorer immunotherapy response.

GSEA was performed on the DEGs identified between Clusters B and A and revealed significant enrichment of 198 GO pathways. Cluster B showed positive enrichment for cell cycle-related pathways, including chromosome organization, nuclear chromosome segregation, and meiosis I cell cycle process. Conversely, pathways negatively enriched in Cluster B were predominantly immune-related, including antigen processing and presentation of peptide antigen, and interferon-gamma production (Figure 3b and Supplementary Table S7). 

Additionally, to identify pharmacologically actionable interactions (gene set source that relates drugs/compounds and their target genes), we cross-referenced the 113 DEGs (Cluster B vs. A comparison) with curated drug-gene interactions from the DSigDB Drug signature database [36]. Notably, the results (Supplementary Table S8) revealed enrichment for 76 drugs/compounds, of which the two candidates with the smallest *p*adj were simvastatin (*p*adj = 2.16 × 10^−9^) and tamibarotene (*p*adj = 2.65 × 10^−7^). 

### 3.7. Immunosuppressive Tumor Microenvironment Defines Cluster B

**Figure 4:**
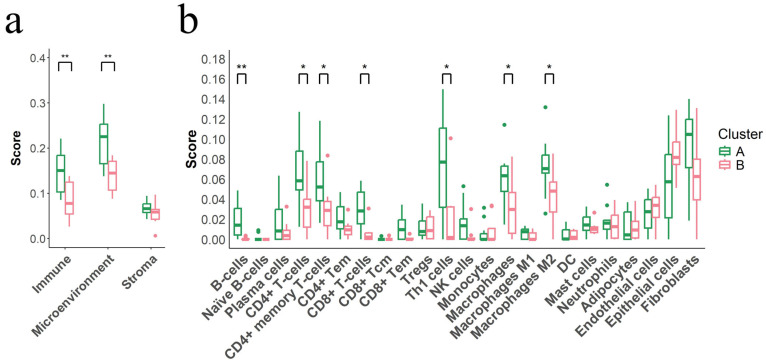
Immune, microenvironment, and stroma scores in Cluster A vs. Cluster B: (**a**) Boxplots illustrating differences in immune, microenvironment, and stroma scores between Cluster A and Cluster B in patients with advanced unresectable melanoma. Scores were derived from RNA expression data using the xCell algorithm. Colored dots represent individual outlier values. Statistical significance is represented by asterisks (* *p* < 0.05, ** *p* < 0.01). (**b**) Boxplots highlighting significant immune cell populations that differ between Cluster A and Cluster B. Immune populations were quantified using xCell and assessed using statistical tests, with significance levels indicated as in (**a**).

The tumor microenvironment (TME) analysis revealed significant differences in immune cell populations between clusters. Cluster B, associated with poorer immunotherapy response, exhibited significantly lower immune (*p* = 0.0093) and microenvironment scores (*p* = 0.0036) compared to Cluster A, while no significant differences were observed in stroma scores (*p* = 0.2401) (Figure 4a and Supplementary Table S9). Specifically, Cluster B showed marked reductions in T-cell subsets, including CD4+ (*p* = 0.0165), CD8+ (*p* = 0.0211), Th1 (*p* = 0.0176), and CD4+ memory T-cells (*p* = 0.0407), as well as decreases in B cells (*p* = 0.0015), macrophages (*p* = 0.0436), and M2 macrophages (*p* = 0.0317) (Figure 4b and Supplementary Table S9).

### 3.8. Distinct Peripheral T-Cell Subpopulations Associate with Immunotherapy Response in Patients with Advanced Melanoma

Cytometry by time-of-flight (CyTOF) analysis of PBMC samples from 17 advanced melanoma patients—seven (41.2%) responders and ten (58.8%) non-responders—was performed to explore peripheral immune profiles. Using FlowSOM and UMAP for clustering and dimensionality reduction, major immune populations, including T-cells, B cells, NK cells, and myeloid cells, annotated by canonical and activation/differentiation markers were identified (Supplementary Figure S6A). A heatmap of median-scaled marker expression across all populations provided a comprehensive overview (Supplementary Figure S6B), showing no significant differences between responders and non-responders groups at the level of main immune subsets (Supplementary Figure S6C, Supplementary Tables S10 and S11). 

**Figure 5:**
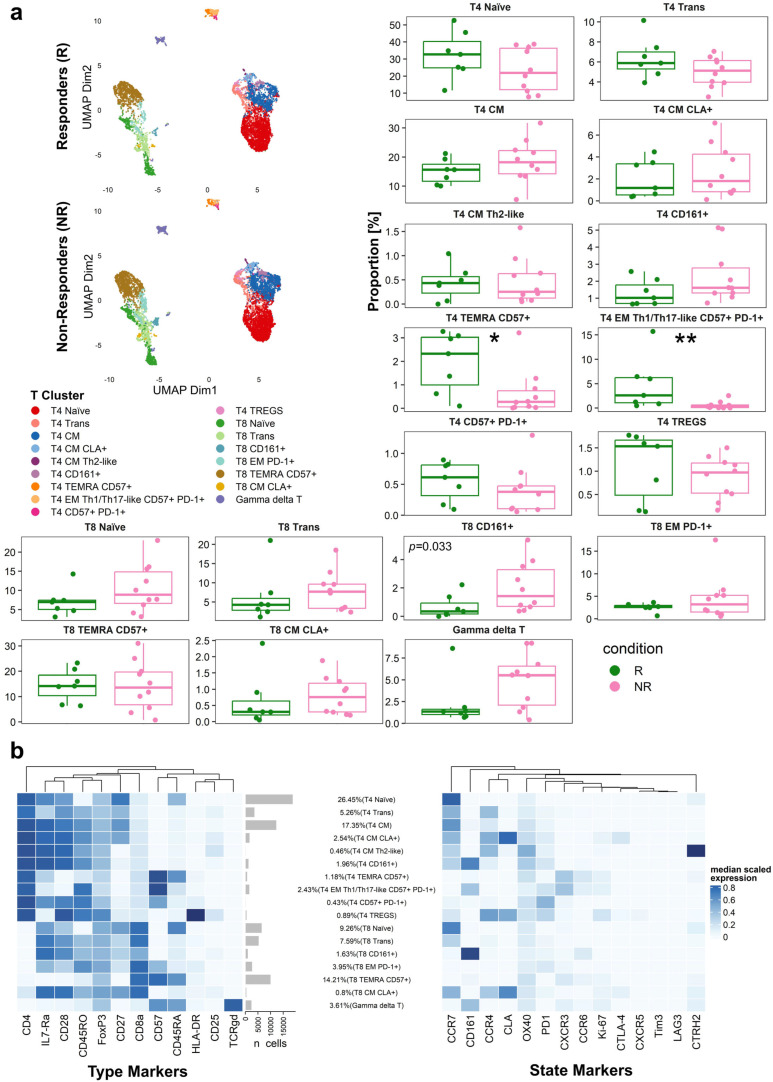
Uniform manifold approximation and projection (UMAP), boxplots, and heatmap visualization of T-cells subsets identified by Flow Self-Organizing Map (FlowSOM) clustering. (**a**) UMAP and boxplots of T-cells subsets. FlowSOM clustering was applied to T-cell data and plotted in two dimensions. Each color denotes a distinct immune population, and the x- and y-axes represent UMAP dimensions 1 and 2, respectively. Boxplots display the frequencies of T-cell populations stratified by responder (R) and non-responder (NR) status. Each boxplot represents the proportion (%) of a specific T-cell subset, with individual points corresponding to individual samples. The horizontal line within each box denotes the median proportion for the respective population. Statistical comparisons were performed by Wilcoxon rank-sum test (* *p* < 0.05, ** *p* < 0.01); (**b**) heatmap of median marker intensities for immune populations identified by FlowSOM clustering. Each row represents a distinct immune subset annotated by canonical and activation/differentiation markers, and each column corresponds to a specific marker. The color scale indicates the median-scaled expression of each marker in each population, aggregated across all samples. Percentages denote the proportion of each immune subset.

Subsequent deeper analysis of T-cells revealed 18 distinct subpopulations (Figure 5a). The median-scaled expression of markers within these subpopulations highlighted their unique phenotypes (Figure 5b). 

Notably, two specific T-cell subpopulations were significantly more abundant in responders compared to non-responders: T4 EM Th1/Th17-like CD57+ PD-1+ (*p* = 0.0068), and T4 TEMRA CD57+ (*p* = 0.0330) (Figure 5a, Supplementary Tables S12 and S13). Conversely, T8 CD161+ cells were more abundant in non-responders (*p* = 0.0330). The clustering analysis of B cells (Supplementary Figure S7A,B, and Supplementary Tables S14 and S15) did not reveal significant differences, whereas the clustering analysis of monocytes and dendritic cells highlighted a significant difference in cluster 6 (*p* = 0.044) (Supplementary Figure S8A,B and Supplementary Tables S16 and S17). 

**Figure 6:**
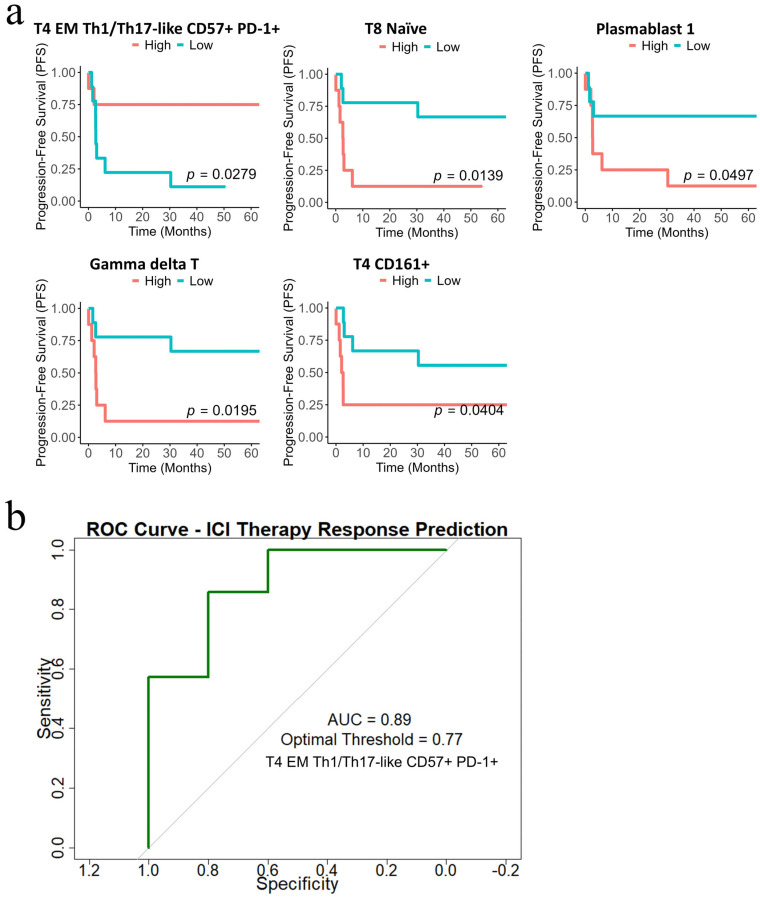
Kaplan–Meier survival plots and receiving-operating characteristic (ROC) curve analysis: (**a**) Kaplan–Meier survival curves for immune subsets identified as significant in the log-rank test. Curves illustrate progression-free survival in months, comparing patients with high (≥median) versus low (<median) frequencies of the subsets. The y-axis indicates the probability of remaining progression-free, while the x-axis represents time in months. Significance was determined using log-rank testing; (**b**) ROC curve illustrating the predictive capacity of the selected immune cell subset in predicting immunotherapy response.

Log-rank survival analysis demonstrated that higher abundance of T4 EM Th1/Th17-like CD57+ PD-1+ subset was significantly associated with improved PFS (*p* = 0.0279) (Supplementary Table S18). In contrast, lower abundance of T4 CD161+ (*p* = 0.0404), T8 Naïve (*p* = 0.0139), Gamma delta T-cells (*p* = 0.0195) and Plasmablast-1 (*p* = 0.0497) were associated with better outcomes (Figure 6a). The ROC analysis confirmed the predictive potential of T4 EM Th1/Th17-like CD57+ PD-1+ cells, with an AUC of 0.893 and an optimal response-predictive threshold of 0.77 (Figure 6b). 

## 4. Discussion

This study explores the molecular and immune landscape underlying response to ICIs in advanced unresectable melanoma, revealing key markers and pathways influencing PFS through an integrative analysis of tumor-intrinsic and microenvironmental factors using high-throughput RNA profiling and mass cytometry. 

The identification of molecular clusters (A and B) highlights significant heterogeneity in the TME and its impact on immunotherapy outcomes. Notably, this study primarily focused on primary tumors, suggesting that immune signatures associated with response may be imprinted years before treatment, highlighting the importance of pre-existing immune profiles. The multivariate Cox model demonstrated that molecular cluster designation is a strong predictor of PFS, and may add predictive information to traditional clinical parameters such as staging at immunotherapy initiation. Cluster B, associated with poorer PFS, exhibited HRs significantly higher than those attributed to staging alone, indicating its potential clinical value for patient stratification and guiding personalized treatment strategies. However, due to the small sample size, definitive conclusions about staging could not be drawn. 

DEA provided evidence for a contribution of tumoral immune suppression and cell cycle dysregulation in shaping the ICI response. Cluster B showed positive enrichment of cell cycle pathways and negative enrichment of immune-related pathways, consistent with prior research suggesting that dysregulated cell cycle activity may drive tumor proliferation and resistance to ICIs [37]. Conversely, the reduced expression of antigen presentation and IFN-γ pathways in Cluster B may reflect an immunosuppressive phenotype, limiting the efficacy of T-cell-mediated anti-tumor responses [11,17]. IFN-γ signaling is critical for enhancing antitumor immunity through improved antigen presentation and T-cell recruitment [15,20]. A complementary drug–gene interaction analysis highlighted simvastatin and tamibarotene with potential to target the DEGs identified in Cluster B. Simvastatin has shown antiproliferative, pro-apoptotic, and cell-cycle arrest effects in melanoma cell lines and reduces tumor growth and metastasis in animal models [38,39,40,41,42,43]. Tamibarotene, a synthetic retinoid acid receptor (RAR)α/β-selective agonist developed to overcome all-trans retinoic acid (ATRA) resistance, induces RAR/retinoid X receptor (RXR)-mediated differentiation and tolerability in relapsed/refractory acute promyelocytic leukemia (APL) [44], but has not yet been studied in melanoma. Moreover, the TME analysis revealed that Cluster B had a significantly lower immune score and reduced levels of key immune cell subsets, including CD4+ and CD8+ T-cells, Th1 cells, and memory T-cells. The depletion of these immune populations is consistent with an immune-desert TME, potentially contributing to poor immunotherapy outcomes in Cluster B. These findings align with previous studies demonstrating that the abundance and functionality of TILs are markers of ICI efficacy [9].

Additionally, the mass cytometry analysis of pre-treatment PBMCs identified specific immune cell subsets correlating with treatment response. Higher abundances of T4 EM Th1/Th17-like CD57+ PD-1+ and T4 TEMRA CD57+ cells were associated with improved PFS, underscoring their potential as markers of immunotherapy response. Notably, elevated circulating PD-1+ CD8+ T-cells has been observed in responders just before initiating treatment, suggesting a broader role for PD-1+ T-cells in predicting therapeutic outcomes [27]. This aligns with studies linking T-cell reinvigoration and effector memory phenotypes to better immunotherapy responses [28]. These results highlight the potential of PD-1+ T-cell subpopulations as markers of immunotherapy response. Log-rank analysis revealed that lower frequencies of CD8+ naïve T-cells associated with improved immunotherapy outcomes, consistent with previous studies showing reduced baseline CD8+ naïve T-cell levels in responders [45,46]. Additionally, higher frequencies of CD4+ CD161+ T-cells were linked to poorer PFS, supporting their proposed immunoregulatory roles [47]. 

The identification of immune suppression and cell cycle dysregulation in Cluster B highlights the need for combination therapies targeting these pathways. Modulating the cell cycle, enhancing antigen presentation, or addressing immune suppression hold promise for improving patient outcomes. Moreover, the potential of peripheral blood profiling as a complementary tool to tumor biopsy data offers a valuable approach for identifying markers of immunotherapy response prior to treatment initiation, enabling more tailored therapeutic decisions.

This study has several limitations, including a relatively small cohort size, which may impact the generalizability, and the use of the HTG EdgeSeq Precision Immuno-Oncology panel. Though effective for profiling oncogenic and immune-related genes in FFPE samples, it lacks the comprehensive coverage of whole transcriptome sequencing. Moreover, further validation using immunohistochemistry or other protein-based methods would strengthen the experimental findings. Future multi-center studies should validate these findings in larger cohorts and expand molecular analyses to ensure broader applicability.

## 5. Conclusions

This study highlights the potential role of molecular and immune profiling in predicting response to ICIs in advanced melanoma. The identification of distinct molecular clusters underscores significant heterogeneity within the TME, highlighting that immune-cold tumor clusters are associated with poorer immunotherapy responses. PD-1+ T-cell subpopulations emerged as potential markers of ICI response, suggesting their value in improving patient stratification. Despite limitations, these findings demonstrate the utility of pre-treatment profiling to guide therapeutic decisions and emphasize the need for validation in larger, multi-center studies to refine immunotherapy strategies.

## Figures and Tables

**Table 1:** Clinical and pathological characteristics of patients treated with immunotherapy by molecular clusters.

Characteristics	Cluster A (*n* = 11)	Cluster B (*n* = 8)	*p*-Value
**Sex**, *n* (%)			1
Females	5 (45.5%)	3 (37.5%)	
Males	6 (54.5%)	5 (62.5%)	
**Age at diagnosis** (years), mean (SD) ^a^	60.2 (17.2)	73.6 (10.5)	0.068
**Age at start of IMT** ^b^ (years), mean (SD)	62.5 (17.4)	75.6 (9.1)	0.072
**Staging ^c^ at diagnosis**			0.898
I–II	5 (45.5%)	1 (12.5%)	
III–IV	6 (54.5%)	7 (87.5%)	
**Staging at start of IMT**			0.307
III	2 (18.2%)	1 (12.5%)	
IV	9 (81.8%)	7 (87.5%)	
**Mitotic Index** ^d^, median (IQR) ^e^	7 (6)	6.5 (4.75)	1
**PFS** ^f^, median (IQR)	59.4 (42.2)	2.4 (7.7)	0.002
**Response** ^g^, *n* (%)			0.003
Responder	8 (72.7%)	0 (0%)	
Non-responder	3 (27.3%)	8 (100%)	
**irAEs** ^h^, *n* (%)			0.058
Yes	9 (81.8%)	6 (75%)	
No	2 (18.2%)	2 (25%)	

^a^: SD, standard deviation; ^b^: IMT, immunotherapy; ^c^: stage using American Joint Committee on Cancer (AJCC) Staging, 8th edition; ^d^: Mitotic Index, number of mitoses per mm^2^; ^e^: IQR, interquartile range; ^f^: PFS, progression-free survival; ^g^: response, defined by the Response Evaluation Criteria in Solid Tumors (RECIST); ^h^: irAEs, immune-related adverse events.

## Data Availability

The authors confirm that the data supporting the findings of this study are available within the article and its supplementary materials, including the log_2_(CPM) values (Supplementary Table S19). Mass cytometry FCS files are available in the Zenodo repository (https://zenodo.org/records/15084569; accessed 6 June 2025).

## Supplementary Materials

The following supporting information can be downloaded at https://www.mdpi.com/article/10.3390/cancers17122022/s1, Figure S1: Timepoints of Biopsies; Figure S2: Gating strategy using Gaussian parameters for quality control and event selection; Figure S3: Kaplan–Meier survival plots for the 10 genes with the lowest *p*-values from log-rank analysis; Figure S4: Kaplan–Meier curves comparing progression-free survival (PFS) between patients in subcluster A1 and subcluster B1; Figure S5: Bootstrap analysis of hierarchical clustering; Figure S6: UMAP, heatmap and boxplot visualization of major immune subsets of peripheral blood mononuclear cells (PBMC); Figure S7: Characterization of circulating B cell populations identified by FlowSOM clustering; Figure S8: Characterization of circulating monocytes and dendritic cell populations identified by FlowSOM clustering; Table S1: Patient, sample data and histopathologic features; Table S2: Gene nomenclature equivalences and eligibility; Table S3: CyTOF Panel Design; Table S4: Clinical and Demographic Characteristics of Patients with Advanced Melanoma Treated with Immune Checkpoint Inhibitors; Table S5: Log-rank analysis; Table S6: Differential Expression Analysis Cluster B vs. Cluster A; Table S7: Gene Set Enrichment Analysis Cluster B vs. Cluster A; Table S8: Drug-gene interaction analysis; Table S9: Cell signatures and statistics Cluster A vs. Cluster B; Table S10: Cluster abundances main populations; Table S11: Statistical analysis main populations; Table S12: Cluster abundances T-cells; Table S13: Statistical analysis T-cells; Table S14: Cluster abundances B cells; Table S15: Statistical analysis B cells; Table S16: Cluster abundances monocytes and dendritic cells; Table S17: Statistical analysis monocytes and dendritic cells; Table S18: Log-rank analysis immune populations; Table S19: log_2_(CPM) values.

## Abbreviations

The following abbreviations are used in this manuscript:

ACD	Acid-citrate-dextrose
AJCC	American Joint Committee on Cancer
ALM	Acral lentiginous melanoma
ANM	Acral nodular melanoma
APL	Acute promyelocytic leukemia
ATRA	All-trans retinoic acid
AUC	Area under the curve
BP	Biological Process
CI	Confidence interval
CPM	Counts per million
CR	Complete response
CyTOF	Cytometry by time-of-flight
dds	DESeqDataSet
DEA	Differential expression analysis
DEGs	Differentially expressed genes
EM	Effector memory
FCS	Flow Cytometry Standard
FFPE	Formalin-fixed, paraffin-embedded
FlowSOM	Flow Self-Organizing Map
GO	Gene Ontology
GSEA	Gene set enrichment analysis
H&E	Hematoxylin and eosin
HCA	Hierarchical clustering analysis
HCB	Hospital Clinic of Barcelona
HIV	Human immunodeficiency virus
HR	Hazard ratio
ICI	Immune-checkpoint inhibitor
IFN-γ	Interferon gamma
IMT	immunotherapy
IQR	Interquartile range
irAEs	Immune-related adverse events
MDIPA	Maxpar Direct Immune Profiling Assay
MLM	Mucosal lentiginous melanoma
MSigDB	Molecular signatures database
NM	Nodular melanoma
NR	Non-responder
*p*adj	Adjusted *p*-value
PBMCs	Peripheral blood mononuclear cells
PC1	Principal component 1
PC2	Principal component 2
PCA	Principal component analysis
PD	Progressive disease
PEI	Preliminary expanded immune signature
PFS	Progression-free survival
PR	Partial response
QC	Quality-control
R	Responder
RAR	Retinoic acid receptor
RECIST	Response Evaluation Criteria in Solid Tumors
ROC	Receiver-operating characteristic
RXR	Retinoid X receptor
SSM	Superficial spreading melanoma
TILs	Tumor-infiltrating lymphocytes
TME	Tumor microenvironment
UMAP	Uniform manifold approximation and projection
