# Supplementary material for: Integrative Molecular and Immune Profiling in Advanced Unresectable Melanoma: Tumor Microenvironment and Peripheral PD-1+ CD4+ Effector Memory T-Cells as Potential Markers of Response to Immune Checkpoint Inhibitor Therapy
Source: Cancers (Basel). 2025 Jun 17;17(12):2022. doi: 10.3390/cancers17122022 (PMC12190280; doi:10.3390/cancers17122022)
Supplement: Supplementary file 1 [file cancers-17-02022-s001.zip › Supplementary Information.pdf]

Article

Integrative Molecular and Immune Profiling in Advanced Unresectable Melanoma: Tumor Microenvironment and Peripheral PD-1+ CD4+ Effector Memory T Cells as Potential Markers of Response to Immune Checkpoint Inhibitor Therapy

Manuel Molina-García [1,2,3,†], María Jesús Rojas-Lechuga [4,†], Teresa Torres Moral [1,3,5], Francesca Crespí-Payeras [1,2,3,5], Jaume Bagué [1,2,3], Judit Mateu [1,3], Nikolaos Paschalidis [6], Vinícius Gonçalves de Souza [7], Sebastian Podlipnik [1,2,3], Cristina Carrera [1,2,3,5], Josep Malvehy [1,2,3,5], Rui Milton Patricio da Silva-Júnior [1,3,8,‡], and Susana Puig [1,2,3,5,‡,*]

[1] Institut d'Investigacions Biomèdiques August Pi I Sunyer (IDIBAPS), 08036 Barcelona, Spain; manmolina@recerca.clinic.cat (M.M.-G.); tetorres@recerca.clinic.cat (T.T.M.); fjcrespi@recerca.clinic.cat (F.C.-P.); bague@recerca.clinic.cat (J.B.); mateu@recerca.clinic.cat (J.M.); podlipnik@clinic.cat (S.P.); ccarrera@clinic.cat (C.C.); jmalvehy@clinic.cat (J.M.); patricioda@recerca.clinic.cat (R.M.P.d.S.-J.)

[2] Facultat de Medicina i Ciències de la Salut, Universitat de Barcelona (UB), c.Casanova, 143, 08036 Barcelona, Spain

[3] Melanoma Unit, Dermatology Department, Hospital Clinic of Barcelona, 170 Villarroel, 08036 Barcelona, Spain.

[4] Otorhinolaryngology Department, Hospital Clínic de Barcelona, CIBERES, IDIBAPS, Universitat de Barcelona, 08036 Barcelona, Spain; mrojas@clinic.cat

[5] Centre of Biomedical Research on Rare Diseases (CIBERER), Instituto de Salud Carlos III, 28029 Madrid, Spain

[6] Biomedical Research Foundation, Academy of Athens (BRFAA), 4 Soranou Efesiou St., 11527 Athens, Greece; npaschal@bioacademy.gr

[7] Molecular Oncology Research Center, Barretos Cancer Hospital, Barretos 14784-400, São Paulo, Brazil; vinicius.gs2110@edu.hospitaldeamor.com.br

[8] Department of Cell and Molecular Biology, Faculty of Medicine of Ribeirão Preto, University of São Paulo (FMRP-USP), Ribeirão Preto 14049-900, São Paulo, Brazil

* Correspondence: spuig@clinic.cat

† These authors contributed equally to this work and share first-author responsibilities.

‡ These authors contributed equally as senior authors.

S2. Materials and Methods

S2.1 Study design, Patients, and Inclusion Criteria

This prospective study was performed at the Hospital Clinic of Barcelona (HCB) in accordance with established ethical guidelines and was approved by the HCB Ethics Committee (approval number HCB/2018/1074).

Patients with advanced, unresectable melanoma who were eligible for immunotherapy (anti-PD-1 alone or in combination with anti-CTLA-4) were included. Inclusion criteria included providing signed informed consent and being a candidate for ICI based on standard clinical guidelines. Exclusion criteria comprised prior ICI treatment, concomitant autoimmune disorders requiring immunosuppressive therapy, active infections (e.g., HIV, hepatitis B/C), or the unavailability of both a formalin-fixed, paraffin-embedded (FFPE) tumor biopsy obtained before ICI initiation and a peripheral blood sample collected pre-ICI. FFPE primary tumors were analyzed; in cases where multiple primary tumors were available, the specimen with the largest Breslow depth was selected, and if the primary tumor was not accessible, the first metastasis obtained before ICI was analyzed. In parallel, peripheral blood mononuclear cells (PBMCs) were prospectively collected before the initiation of immunotherapy. The timepoint of biopsies is shown in **Supplementary Figure S1**. The initiation date of immunotherapy served as the index date for progression-free survival (PFS) analysis, calculated from the start of treatment to either the last follow-up date (December 1, 2024) or the date of documented disease progression, as determined by the Response Evaluation Criteria in Solid Tumors (RECIST)[1] obtained from medical records. Patients were considered to have progressed if they met these criteria at any time after immunotherapy initiation.

Demographic and clinical data, as well as disease status, were retrieved from medical records. Histopathological parameters included ulceration status, mitotic index, and BRAF mutations (**Supplementary Table S1**).

Melanoma staging followed the American Joint Committee on Cancer (AJCC) 8th edition guidelines for melanoma of the skin.[2] Additional histopathological evaluations were performed on hematoxylin and eosin (H&E)-stained slides digitized by the Hamamatsu NanoZoomer S60 Digital Pathology Image scanner (resolution of 440 nm per pixel). The resulting digital images were then assessed using NDPI.view2 software (version 2.9.29; Hamamatsu Photonics).

S2.2 Tumor sample preparation, library construction, sequencing and data processing

Tumor samples were collected during surgical procedures and subsequently subjected to thorough histopathological assessment and immunohistochemical assays, targeting key melanocytic markers alongside proliferation and aggressiveness indicators. H&E staining was used to clearly demarcate the tumor regions. For molecular analysis, FFPE tissue blocks were cut into 5 μm sections. Unstained slides were processed by HTG EdgeSeq technology, focusing on a macrodissected tumor region of 12 mm^2 to 30 mm^2. Quantitative mRNA expression profiling was performed on these macrodissected samples. All procedures for sample handling, library preparation, and sequencing were carried out as per the manufacturer's guidelines.

To facilitate mRNA capture in FFPE samples, tissues were permeabilized with HTG lysis buffer, followed by the addition of gene-specific Nuclease Protection Probes (NPP) from the Precision Immuno-Oncology Panel (comprising 1392 probes; HTG, Tucson, AZ), thereby forming stable RNA-probe heteroduplexes. Non-hybridized mRNA and excess NPPs were digested with S1 nuclease, and PCR-based barcoding was then performed. Sequencing was conducted on the Illumina NextSeq 550 platform (Illumina, San Diego, CA). FASTQ files generated by the sequencing instrument were parsed and mapped to the probe list using the HTG Parser (HTG, Tucson, AZ). Quality control parameters included positive control read counts (QC0), total read counts per sample (QC1), and standard deviation between case probe read counts (QC2).

S2.3 Survival analysis

Survival analysis using the log-rank test was performed to evaluate the relationship between gene expression levels and progression-free survival (PFS). Patients were divided into high and low expression groups based on the median CPM values for each gene, and Kaplan-Meier curves were generated to compare PFS between these groups. The log-rank test was then applied, using PFS in months as the time variable and progression as the event. Genes exhibiting significant associations ($P < 0.05$) were visualized using Kaplan-Meier plots. All p-values, including those from non-significant genes, were exported for comprehensive downstream analysis.

Cox proportional hazards models were used to assess the association between clinical and molecular factors and PFS. First, a univariate analysis was conducted to evaluate the effect of each variable independently. Subsequently, a multivariate Cox proportional hazards model was performed to adjust for potential confounders and identify independent predictive factors for immunotherapy response. The models were fitted using the coxph() function in R, with robust

standard errors. Survival time was recorded in months, and the event status was coded as 0 for censored patients and 1 for progression of disease.

Forest plots were generated using the forest model package built on ggplot2 (version 3.5.1) to illustrate hazard ratios (HRs) and 95% confidence intervals (CI). All analyses – including Kaplan-Meier curve generation, log-rank testing, Cox proportional hazards modeling, and graphical visualization – were conducted in R. Specifically, the survival package (version 3.7.0) supported log-rank tests (survdiff) and Cox models (coxph), while the survminer package (version 0.4.9) was utilized for Kaplan-Meier plot creation. Forest plots were generated with ggplot2 (version 3.5.1). Statistical significance was established at $P < 0.05$.

S2.4 Hierarchical clustering analysis (HCA)

Gene expression raw counts (RC) from 55 genes with significant log-rank p-values for PFS were used. First, a DESeqDataSet (dds) object was created, and counts per million (CPM) values were computed. Subsequently, log2CPM transformation was performed, and robust Z-scores were calculated to normalize expression levels across samples. HCA of the samples was conducted using Pearson correlation with average linkage, while the probes were clustered based on Manhattan distance followed by Ward.D linkage. The ComplexHeatmap package (version 2.15.4) was used to generate a heatmap featuring color-coded clinical annotations. A PCA was also conducted on an rlog-transformed dataset to stabilize variance, using the plotPCA function from DESeq2 in combination with ggplot2 to visualize sample separation according to cluster assignments. Finally, a bootstrap approach using pvclust was performed on the Z-score matrix to assess cluster stability under N = 1000 replicates.

S2.5 Differential expression analysis

DEA was performed between clusters, using the group with better PFS as the reference. A DESeqDataSet object was created, and differentially expressed genes (DEGs) were identified using the DESeq2 package (v.1.38.3). Genes were considered differentially expressed if they had an adjusted p-value (padj) < 0.05. The padj was computed using the Benjamini-Hochberg method to control for false discovery rate (FDR) in multiple testing. Volcano plots highlighted genes with log_2FoldChange ≥ 1 or ≤ -1 and padj < 0.05 as upregulated or downregulated, respectively.

2.6 Gene set enrichment analysis (GSEA)

GSEA was conducted to identify enriched pathways associated with differential expression between clusters. Probes lacking direct correspondences or those associated with non-unique genes (e.g., detecting multiple isoforms or gene families) were reannotated or excluded; details on gene nomenclature mappings and eligibility are provided in **Supplementary Table S2**. A curated panel of 1,378 genes from differential expression dataset was used to examine enriched biological processes relevant to the experimental conditions. HUGO Gene Symbols were mapped to their corresponding Entrez IDs using the "bitr" function in the "clusterProfiler" package and the "org.Hs.eg.db" annotation database, retaining only valid Entrez IDs. A ranked gene list was then generated based on the test statistics from the differential expression analysis and sorted in descending order of significance. The "gseGO" function in "clusterProfiler" was employed for GSEA under the "BP" (Biological Process) ontology, applying Benjamini-Hochberg (BH) correction with a padj ≤ 0.05.

S2.7 Immune, Stroma, and Tumor Microenvironment signatures

The xCell algorithm[3] (version 1.1.0) was employed to characterize 19 immune and 4 stromal cell populations across clusters. (ref) Enrichment scores were calculated for each cell subtype using the xCellAnalysis() function on normalized counts, incorporating spillover corrections to improve accuracy. Additionally, immune, stroma, and microenvironment scores were determined for every sample. The statistical significance of cell type-specific scores was then evaluated via xCellSignificanceBetaDist(), and adjusted p-values were exported alongside computed scores, for cluster comparisons. Cluster differences were evaluated using t-tests (Student's or Welch's) for normally distributed data and the MannWhitney U test otherwise, with normality and variance homogeneity assessed by Shapiro-Wilk and Bartlett's tests, respectively.

S2.8 Blood sample preparation, PBMCs mass cytometry staining and analysis

The blood was collected in acid-citrate-dextrose (ACD) collection tubes and processed to isolate peripheral blood mononuclear cells (PBMCs) using density gradient centrifugation. Briefly, the blood was centrifuged to separate plasma, and the mononuclear cells were isolated by layering over Ficoll in LeucoSep tubes. The cells were washed, resuspended in freezing medium (FBS + 10% DMSO + 1% penicillin / streptomycin), and cryopreserved at -80ºC in Mr. Frosty containers before transferring to liquid nitrogen for long-term storage. PBMCs were gently thawed in a 37°C water bath

and washed twice in RPMI 1640 supplemented with 10% fetal bovine serum (FBS) to remove dimethyl sulfoxide (DMSO), ensuring a viability of ≥80%. Cells were then blocked with Human TruStain FcX and stained with the Maxpar Direct Immune Profiling Assay (MDIPA, Standard BioTools) plus 10 additional antibodies (the complete panel is provided in **Supplementary Table S3**). Following fixation in 1.6% formaldehyde and nuclear permeabilization, nuclear antigen antibodies were added, and an iridium-based intercalator was used to label DNA. Finally, cells were pelleted, resuspended in the residual volume, and stored at –80°C for subsequent analysis.

Prior to acquisition on the Helios mass cytometer (Standard BioTools, South San Francisco, CA, USA), thawed PBMCs were washed twice with Maxpar Cell Staining Buffer and once with Maxpar Cell Acquisition Solution to ensure optimal sample preparation. Subsequently, cells were mixed with EQ Four Element Calibration Beads (containing 140Ce) to ensure optimal instrument performance and data normalization. Stained cells were run on the Helios mass cytometer using the MDIPA acquisition template in CyTOF software (version 6.7.1016 or higher). Post-acquisition, the data were normalized and exported as FCS files for downstream analysis.

Normalized FCS files were manually pre-processed with bivariate dot plots in FlowJo™ (v10.8 Software, BD Life Sciences) (**Supplementary Figure S2**). Briefly, beads used for normalization were gated out (140 Ce vs Time), followed by gates with gaussian parameters were (Residual, Offset, Center, Width vs Time).[4] Finally, live singlet CD45+ cells were selected and exported as new FCS files for downstream analysis in the R programming environment (version 4.1.0). An R-based pipeline was then employed, integrating FlowSOM (version 2.11.2) and UMAP (uwot, version 0.2.2) within the CATALYST (version 1.26.1) package as outlined in the mass cytometry workflow described by Nowicka et al. (2019) in the cytofWorkflow vignette on Bioconductor.[5] The choice of metaclusters (FlowSOM) considered both computational and biological factors: major immune subsets such as T, B, NK and Myeloid cells were identified, followed by subsetting to uncover detailed subpopulations. For T cells, data were expected to resolve 10-20 distinct cell populations; thus, an over-clustering strategy was applied to avoid excluding potentially significant subpopulations. The ConsensusClusterPlus algorithm was then used to assess the stability and consistency of the clusters. Statistical significance was assessed within R using the Compare_means function with unpaired two-samples Wilcoxon test and Benjamini-Hochberg (BH) multiple testing correction ($p \leq 0.05$). A receiver operating characteristic (ROC) curve analysis was then performed in R using the pROC package (v.1.18.5) to assess its predictive capacity for immunotherapy response. The area under the curve (AUC) optimal abundance threshold using the Youden index were calculated.

S2.9 Statistical Analysis

Continuous variables were assessed for normality using the Shapiro-Wilk test. Normally distributed data were presented as means (standard deviations), while non-normally distributed data were expressed as medians (interquartile ranges, IQRs). Survival times were reported as medians (IQRs). Kaplan-Meier analyses were performed to evaluate PFS across clusters, with differences in survival rates evaluated using the log-rank test. Categorical variables were summarized as frequencies and percentages, then analyzed by Chi-square ($\chi 2$) or Fisher's exact test, as appropriate. All tests were two-tailed, with a 0.05 alpha level for statistical significance. Analyses were conducted using STATA software v.16.1 (StataCorp, TX, USA) and the R language (version 4.2.3) in RStudio (version 2023.06.0+421).

References

1. Eisenhauer EA, Therasse P, Bogaerts J, et al. New response evaluation criteria in solid tumours: Revised RECIST guideline (version 1.1). *Eur J Cancer*. 2009;45(2):228-247. doi:10.1016/j.ejca.2008.10.026
2. Amin M, Edge S, Greene F, et al., eds. *AJCC Cancer Staging Manual*. Vol 7. 8th ed. Springer; 2017.
3. Aran D, Hu Z, Butte AJ. xCell: Digitally portraying the tissue cellular heterogeneity landscape. *Genome Biol*. 2017;18(1):1-14. doi:10.1186/s13059-017-1349-1
4. Bagwell CB, Inokuma M, Hunsberger B, et al. Automated Data Cleanup for Mass Cytometry. *Cytom Part A*. 2020;97(2):184-198. doi:10.1002/cyto.a.23926
5. Nowicka M, Krieg C, Crowell HL, et al. CyTOF workflow: Differential discovery in high-throughput high-dimensional cytometry datasets. *F1000Research*. 2017;6:748. doi:10.12688/f1000research.11622.3

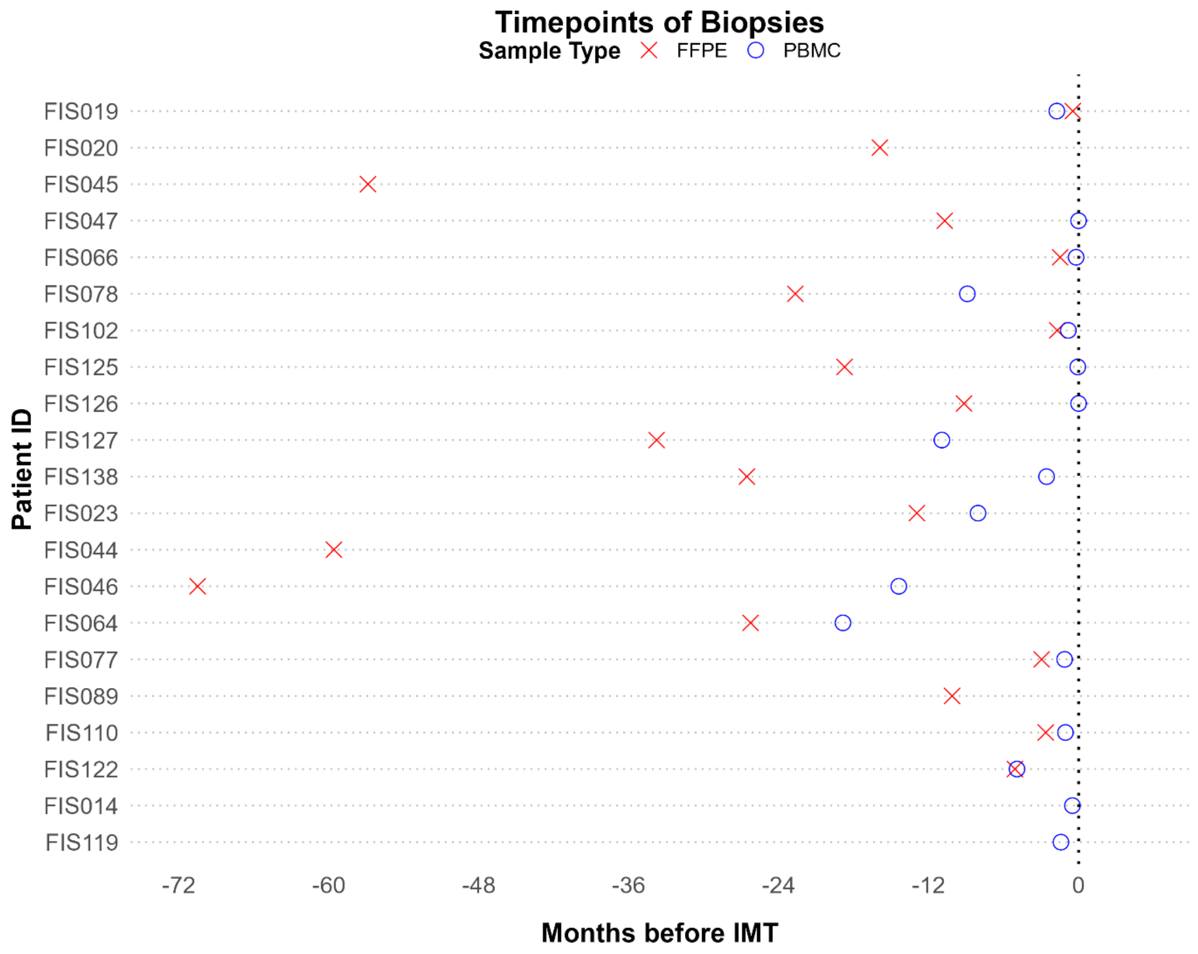

Figure S1. **Timepoints of Biopsies**. This figure illustrates the collection timepoints of tumor biopsies and peripheral blood mononuclear cells (PBMCs) from patients before initiating immune checkpoint inhibitor (ICI) therapy. The x-axis represents the months before ICI initiation, and the y-axis corresponds to individual patients. Formalin-fixed paraffin embedded (FFPE) tumor biopsies are marked with red crosses, while PBMC samples are represented by blue circles. Each dotted horizontal line denotes a single patient. The vertical dotted line at timepoint 0 months indicates the initiation of ICI therapy.

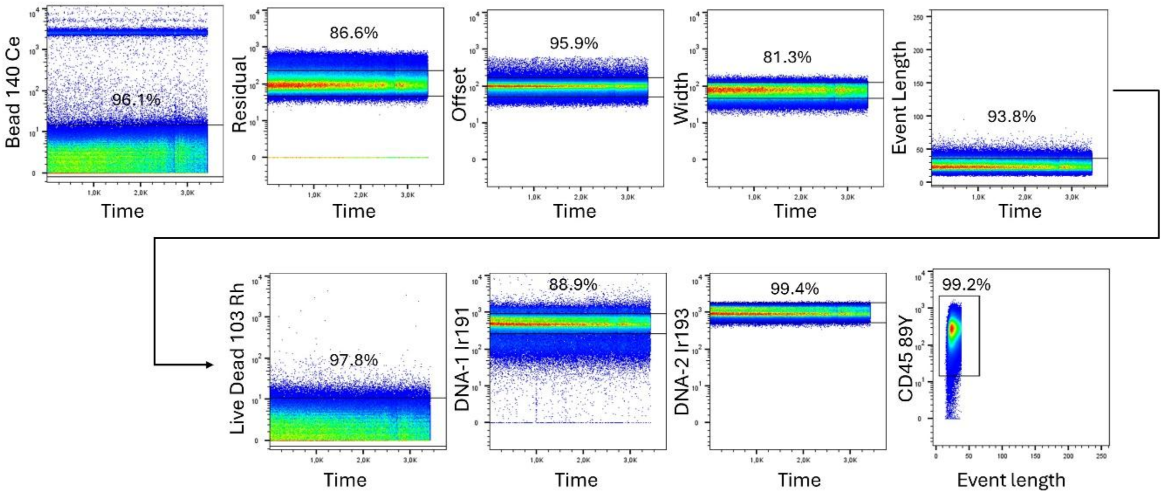

Figure S2. **Gating strategy using Gaussian parameters for quality control and event selection**. The percentages represent the proportion of events retained at each step after data cleanup.

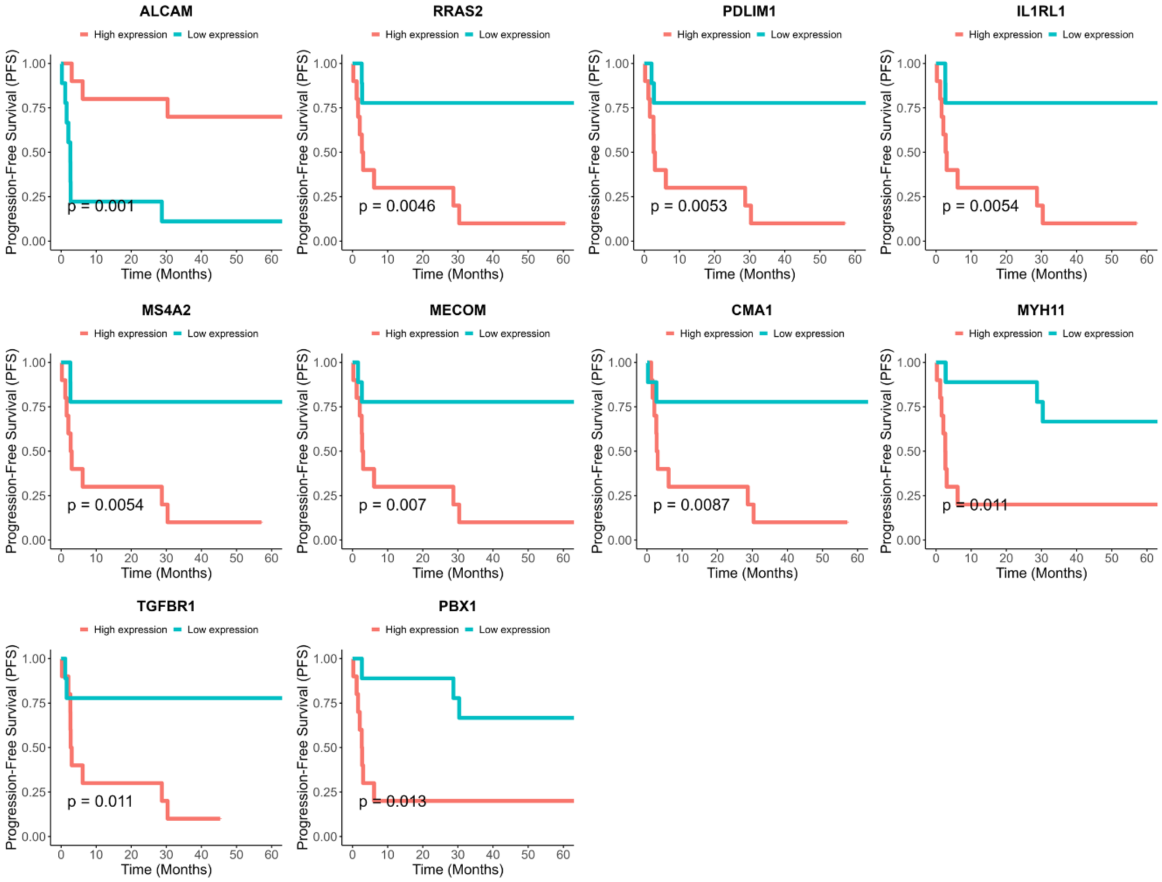

Figure S3. **Kaplan-Meier survival plots for the 10 genes with the lowest P-values from log-rank analysis**. The Kaplan-Meier curves show progression-free survival (PFS) in months, comparing high (≥ median) versus low (< median) expression for each gene. Expression levels were quantified in counts per million (CPM). The y-axis indicates the probability of remaining progression-free, while the x-axis represents time in months.

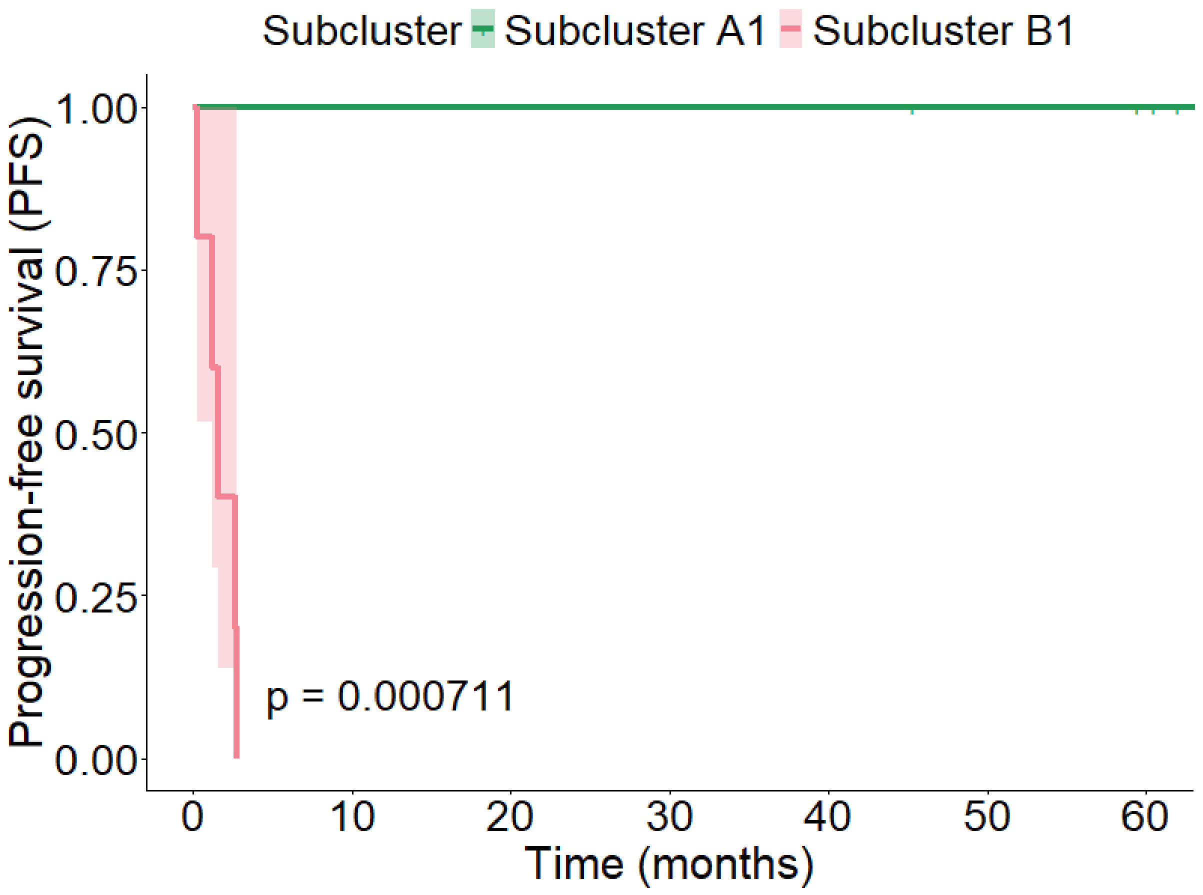

Figure S4. **Kaplan-Meier curves comparing progression-free survival (PFS) between patients in subcluster A1 and subcluster B1**. Shaded areas represent the 95% confidence intervals. The x-axis shows time in months, and the y-axis indicates the probability of remaining progression-free.

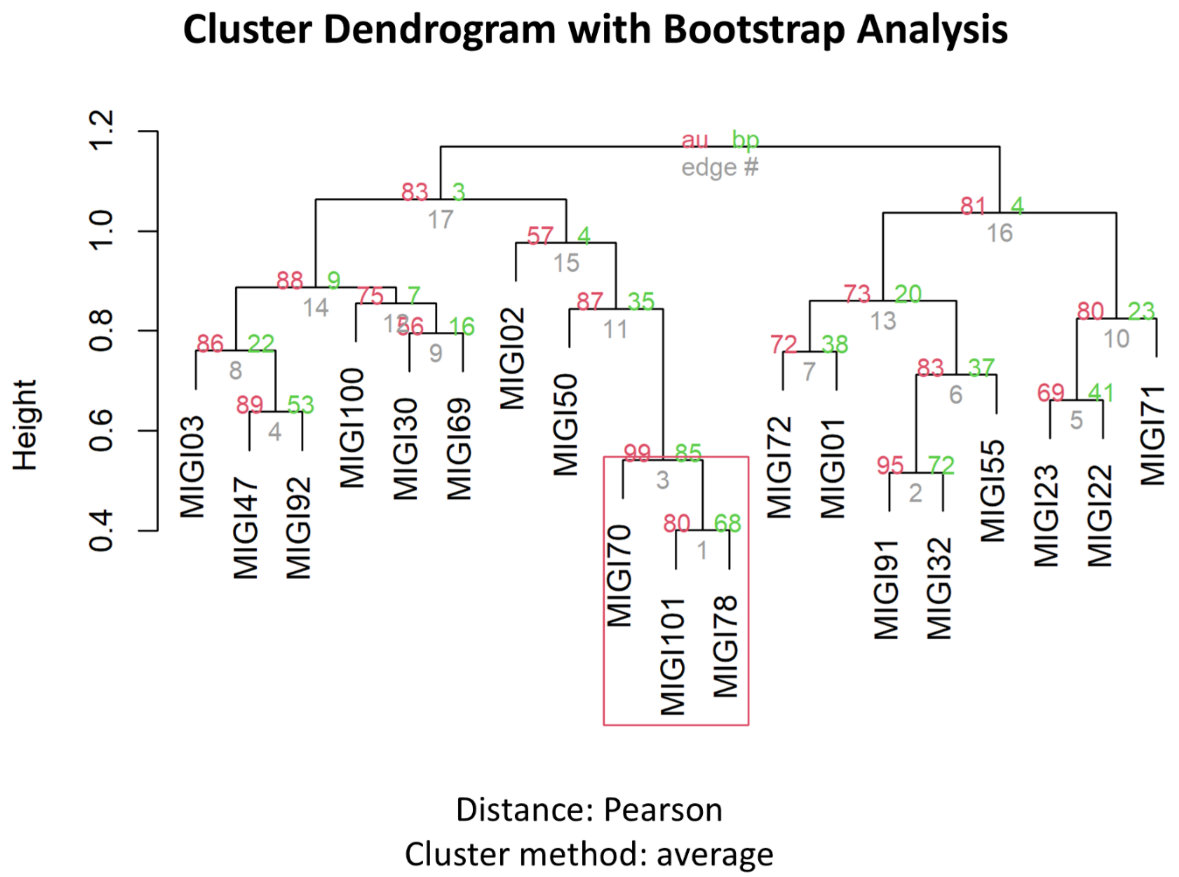

Figure S5. **Bootstrap analysis of hierarchical clustering**. This dendrogram was generated from gene expression profiles derived from robust z-scores, using Pearson correlation distance and average linkage. A 1000-iteration bootstrap analysis (pvclust) was performed to assess the robustness of the resulting clusters, with the approximately unbiased (AU) and bootstrap probability (BP) values indicated at each node. The vertical axis depicts the relative distances among samples, reflecting the degree of similarity in their transcriptomic profiles.

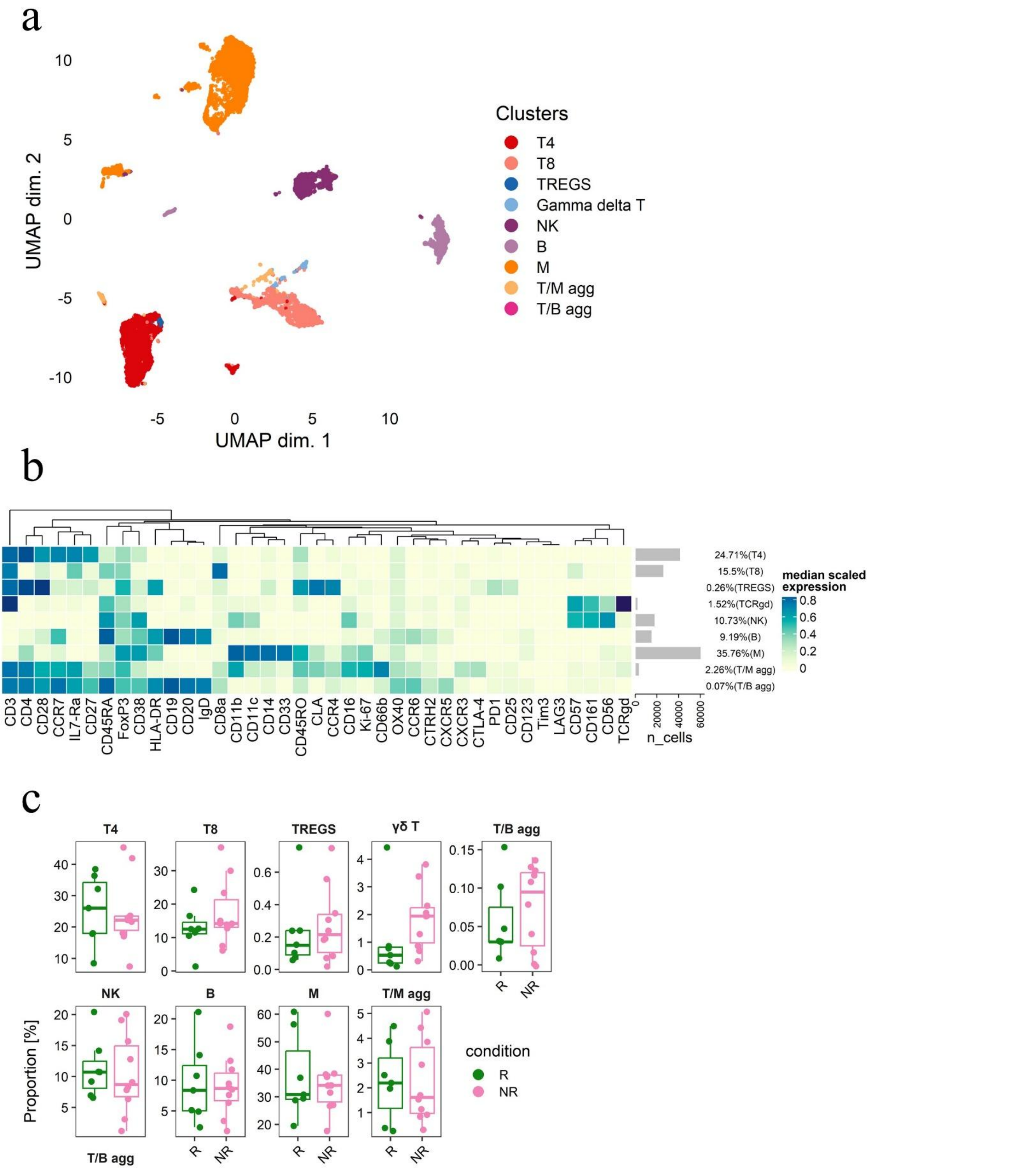

Figure S6. **UMAP, heatmap and boxplot visualization of major immune subsets of peripheral blood mononuclear cells (PBMC)**. **S6a**: Ten thousand PBMC events stained with the 40-marker panel (Supplementary Table S16) was subjected to FlowSOM clustering and plotted in two dimensions. Each color denotes a distinct immune population and the x- and y-axes represent UMAP dimensions 1 and 2, respectively; **S6b**: Heatmap of median marker intensities for immune populations identified by FlowSOM clustering. Each row represents a distinct immune subset annotated by canonical and activation / differentiation markers,

and each column corresponds to a specific marker. The color scale indicates the median-scaled expression of each marker in each population, aggregated across all samples. Percentages denote the proportion of each immune subset; **S6c**: Boxplots showing the frequencies of circulating immune populations identified through FlowSOM clustering using a 40-marker panel, stratified by responder (R) and non-responder (NR) status. Each boxplot represents the proportion (%) of a specific circulating immune population, with individual points corresponding to individual samples. The horizontal line within each box denotes the median proportion for the respective population.

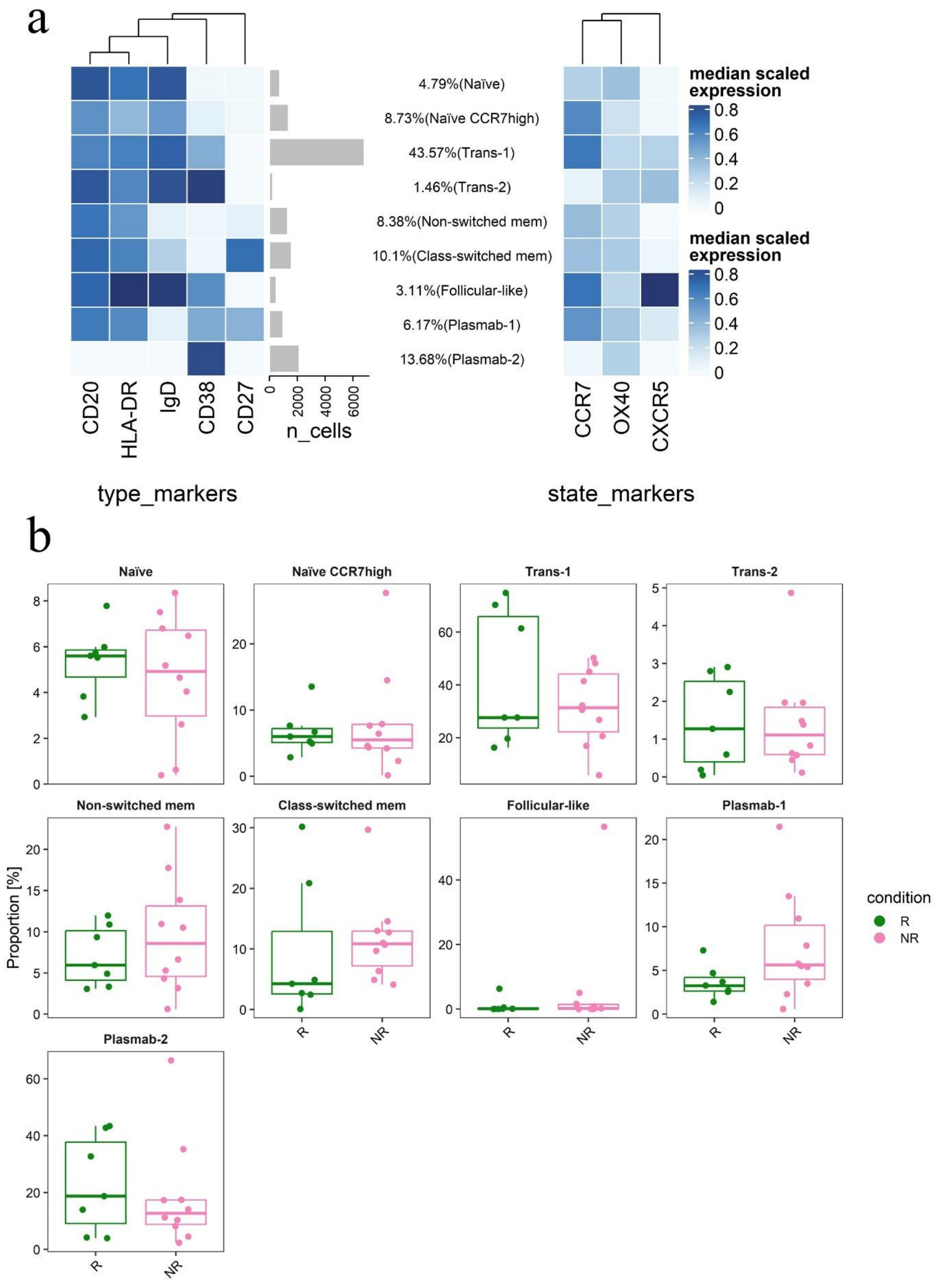

Figure S7. Characterization of circulating B cell populations identified by FlowSOM clustering. S7a: Heatmap of the median marker intensities for B cell populations identified by FlowSOM clustering. Each row represents a distinct B cell subset annotated by canonical and activation / differentiation markers, and each column corresponds to a specific marker. The color scale indicates the median-scaled expression of each marker in each population, aggregated across all samples. Percentages denote the proportion of each B cell subset; **S7b**: Boxplots showing the frequencies of circulating B cells identified through FlowSOM clustering, stratified

by responder (R) and non-responder (NR) status. Each boxplot represents the proportion (%) of a specific B cell subset, with individual points corresponding to individual samples. The horizontal line within each box denotes the median proportion for the respective population.

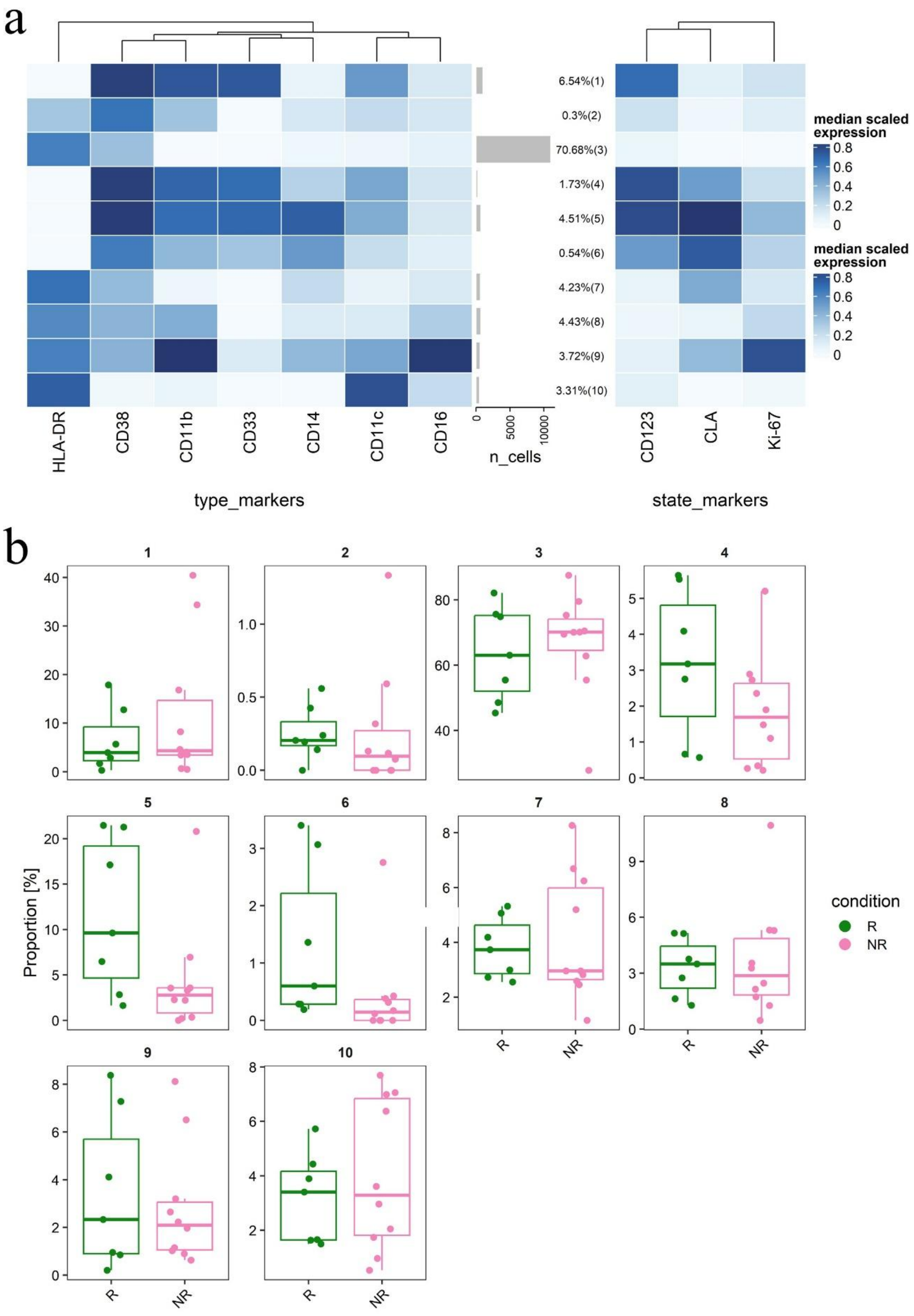

Figure S8. **Characterization of circulating monocytes and dendritic cell populations identified by FlowSOM clustering. S8a**: Heatmap of the median marker intensities for myeloid cell populations identified by FlowSOM clustering. Each row represents a distinct myeloid cell subset annotated by canonical and activation / differentiation markers, and each column corresponds to a specific marker. The color scale indicates the median-scaled expression of each marker in each population, aggregated across all samples. Percentages denote the proportion of each myeloid cell subset; **S8b**: Boxplots showing the frequencies of myeloid cells

including monocytes and dendritic cells identified through FlowSOM clustering, stratified by responder (R) and non-responder (NR) status. Each boxplot represents the proportion (%) of a specific myeloid cell subset, with individual points corresponding to individual samples. The horizontal line within each box denotes the median proportion for the respective population.
