# Supplementary material for: Integrative Molecular and Immune Profiling in Advanced Unresectable Melanoma: Tumor Microenvironment and Peripheral PD-1+ CD4+ Effector Memory T-Cells as Potential Markers of Response to Immune Checkpoint Inhibitor Therapy
Source: Cancers (Basel). 2025 Jun 17;17(12):2022. doi: 10.3390/cancers17122022 (PMC12190280; doi:10.3390/cancers17122022)
Supplement: Supplementary file 1 [file cancers-17-02022-s001.zip › Supplementary Table S4.pdf]

Supplementary Table S4. Clinical and Demographic Characteristics of Patients with Advanced Melanoma Treated with Immune Checkpoint Inhibitors**.**

**Characteristics**	**Patients with Advanced Melanoma (N = 21)**
**Age**[a] (years), mean (SD)[b]	69.1 (15.2)
**Sex**, n (%)	
Male	13 (61.9%)
Female	8 (38.1%)
**Staging**[c], n (%)	
IIIB	1 (4.8%)
IIID	2 (9.5%)
IVM1a	8 (38.1%)
IVM1b	5 (23.8%)
IVM1c	3 (14.3%)
IVM1d	2 (9.5%)
**PFS**[d], median (IQR)	28.7 (56.7)
**BRAF mutations**, n (%)	
Negative	14 (66.7%)
Positive	7 (33.3%)

a: Age at start of immunotherapy; **b**: SD, standard deviation; **c**: Staging at start of immunotherapy using American Joint Committee on Cancer (AJCC) Staging, 8th edition; **d**: PFS, progression-free survival.
